# Clinical and Microbiological Profile of a Retrospective Cohort of Enteric Fever in 2 Spanish Tertiary Hospitals

**DOI:** 10.1097/MD.0000000000000791

**Published:** 2015-05-29

**Authors:** Adrián Sánchez-Montalvá, Ángela Martínez-Pérez, José Antonio Pérez-Molina, Juan José González-López, Rogelio Lopez-Vélez, Fernando Salvador, Irene Sánchez, Anna M. Planes, Israel Molina

**Affiliations:** From the Department of Infectious Diseases, Vall d’Hebron University Hospital, PROSICS Barcelona, Universitat Autònoma de Barcelona, Barcelona (AS-M, FS, IS, IM); Tropical Medicine, Department of Infectious Diseases, Ramón y Cajal University Hospital, IRYCIS, Madrid (AM-P, AP-M, RL-V); and Department of Microbiology, Vall d’Hebron University Hospital, PROSICS Barcelona, Universitat Autònoma de Barcelona, Barcelona, Spain (JJG-L, AMP).

## Abstract

Enteric fever in high-income countries is diagnosed mainly in patients returning from endemic countries. We assess the clinical, microbiological, and prognosis aspects of enteric fever in 2 Spanish tertiary hospitals.

A retrospective observational study was conducted at Vall d’Hebron University Hospital and Ramón y Cajal University Hospital in Spain. We reviewed medical records of all patients who were diagnosed with enteric fever from January 2000 to January 2014 at these hospitals.

We identified 47 patients with enteric fever episodes. According to their travel history, 35 (74.5%) patients had travelled to highly endemic countries. Imported enteric fever was acquired mainly in Asia (70.3%). Imported infections were implicated in travelers (48.6%), visiting friends and relatives (40%) and immigrants (11.4%). We found that 12 patients were diagnosed with enteric fever without a travel history (autochthonous infection). The resistance profile of the isolates showed decreased ciprofloxacin susceptibility in 66.7% of the imported group and 8.3% of the autochthonous group (*P* = 0.001). *Salmonella* strains from patients returning from Asia had an increased risk of having decreased ciprofloxacin susceptibility (odds ratio, 52.25; 95% confidence interval: 8.6–317.7).

Patients with imported enteric fever are at higher risk for having a *Salmonella* strain with decreased ciprofloxacin susceptibility, especially in patients returning from Asia. Initial treatment with third-generation cephalosporin or azithromycin is strongly recommended until a drug-susceptibility test is available. Prevention strategies such as pretravel counseling and immunization before travel may be beneficial.

## INTRODUCTION

*Salmonella enterica* is the causative agent of enteric fever syndrome, a systemic febrile illness with severe consequences if left untreated. The most prevalent enteric fever is typhoid fever produced by *S. enterica* serovar Typhi (*S.* Typhi). Other serotypes, such as *S. enterica* serovar Paratyphi (*S*. Paratyphi) A, B and C, can also cause systemic disease, although with less frequency.^1^

At the present time, enteric fever is rare in high-income countries because of the improvement in sanitation and hygienic conditions. However, it is a major health issue in developing countries. The majority of the 27 million cases of typhoid fever worldwide occur in Africa and Asia.^2^ Patients with enteric fever in non-endemic areas usually have a travel history or close contact with an imported source case. Travelers who visit friends and relatives (VFR) have the highest risk of acquiring the disease among travelers.^3^

Classically, enteric fever has been defined as a systemic illness with 2 stages. Initially, fever appears progressively with headache, bradycardia, sweating, muscle and abdominal pain. Thereafter, defervescence begins spontaneously. The risk of complications such as bleeding, intestinal perforation, and acalculous cholecystitis, among others, is higher in the defervescence stage, but complications can arise at any time during infection.^4^ If untreated, the mortality rate is approximately 5% to 20%.^5,6^

The definitive diagnosis of enteric fever is based on the isolation of the bacteria. Blood culture is positive in up to 80% of the patients. Stool, urine, or rose-spot cultures have less sensitivity.^7^

Chloramphenicol was used as the first-line agent for the treatment of typhoid fever from the 1950s through the 1970s until resistant isolates emerged. To overcome this, trimethoprim–sulphamethoxazole and ampicillin were then employed. However, strains resistant to all three antimicrobials (multidrug-resistant [MDR] strains) emerged during the 1980s and 1990s; as a result, quinolones were used as first-line therapy.^4^ Subsequently, isolates with decreased susceptibility or resistance to ciprofloxacin appeared.^1,8^ Therefore, third-generation cephalosporins are now widely used when resistant strains are suspected.^4^

Our concern is focused on Asia where the majority of infections caused by strains with decreased susceptibility or resistance to ciprofloxacin occur, probably because of the abuse and misuse of antibiotics.^3^

Reports from the 1990s and the beginning of the 21st century highlight the increasing rates of strains with decreased susceptibility to ciprofloxacin. Our hypothesis was that due to the lack of proper and active control strategies the rates of resistant strains would continue to rise in low-income countries.

The objective of our study is to describe the epidemiological, clinical, microbiological, and prognostic characteristics of enteric fever diagnosed in two reference hospital units in Spain, and compare these characteristics between autochthonous and imported infections.

## METHODS

An observational retrospective study was conducted in 2 tertiary Spanish hospitals: Vall d’Hebron University Hospital in Barcelona and Ramón y Cajal University Hospital in Madrid. Both hospitals have an International Health Unit that specializes in attending migrant patients and travelers. Patients from all ages with symptoms consistent with enteric fever and positive *S.* Typhi or *S*. Paratyphi blood and/or stool cultures from January 2000 to January 2014 were included. Data regarding epidemiological, clinical, microbiological, treatment, and prognostic information were retrieved from medical records.

Patients were classified into 4 groups according to origin and travel history: autochthonous, people living in Spain for at least 1 year and without any international travel history in the last 4 weeks before onset of symptoms; travelers, people with travel history within 4 weeks of onset of symptoms; VFR, immigrants returning to their country of origin to visit friends or relatives and having symptoms within the first 4 weeks of their return; and immigrants, people arriving in Spain within 4 weeks of the onset of symptoms.

### Microbiological Data

Blood cultures were performed in a BacT/ALERT 3D system (bioMérieux, Marcy l’Etiole, France) and stool cultures by standard microbiological methods. Isolates identification was performed using the VITEK 2 or API 20E systems (bioMérieux) and serotyping by slide agglutination using commercial antisera according to the Kauffmann–White scheme.

Antimicrobial susceptibility to ampicillin, amoxicillin-clavulanate, piperacillin-tazobactam, cefuroxim, cefoxitin, cefotaxime, ceftriaxone, ceftazidime, cefepime, imipenem, gentamicin, amikacin, nalidixic acid, ciprofloxacin, and trimethoprim-sulfamethoxazole was assessed by disc diffusion in all the isolates following Clinical and Laboratory Standards Institute (CLSI) recommendations.^9^ Minimum inhibitory concentration (MIC) to ciprofloxacin was determined by E-test (bioMérieux) in selected isolates. According to CLSI interpretative criteria, if the MIC to ciprofloxacin was ≥1 μg/mL, the isolate was considered resistant. Reduced susceptibility to ciprofloxacin was defined when the MIC to ciprofloxacin was between 0.125 and 0.99 μg/mL and/or the isolate was resistant to nalidixic acid.

### Ethical Considerations

The study protocol was approved by the Ethical Review Boards of Vall d’Hebron University Hospital (Barcelona, Spain) and Ramón y Cajal University Hospital (Madrid, Spain). Procedures were performed in accordance with the ethical standards laid down in the Declaration of Helsinki as revised in 2000.

### Statistical Analysis

Data were analyzed with IBM^®^ SPSS^®^ Statistics software (v.21.0.0.0; IBM SPSS, Armonk, NY). The median and interquartile range (IQR) were calculated for quantitative variables. Frequencies and percentages were calculated for qualitative variables. Analysis was performed using Student *t* test or Mann–Whitney *U* test for quantitative variables and *χ*^2^ test or Fisher test for qualitative variables when appropriate. Tests were considered significant when the 2-tailed *P* value was <0.05.

## RESULTS

Overall, 47 patients and 48 episodes were identified (1 patient suffered a relapse). Twenty-seven (57.4%) patients were from Vall d’Hebron University Hospital and 20 (42.6%) were from Ramón y Cajal University Hospital. Twenty-nine (61.7%) patients were male. Their median age was 28 years (IQR, 24–41 years). An immunosuppressive condition was present in 3 (6.4%) patients, all of whom had human immunodeficiency virus (HIV) infection. Thirty-five patients (74.5%) reported a travel history to an endemic country within 4 weeks of onset of symptoms. Table 1 shows the baseline characteristics of patients according to travel history.

**TABLE 1:**
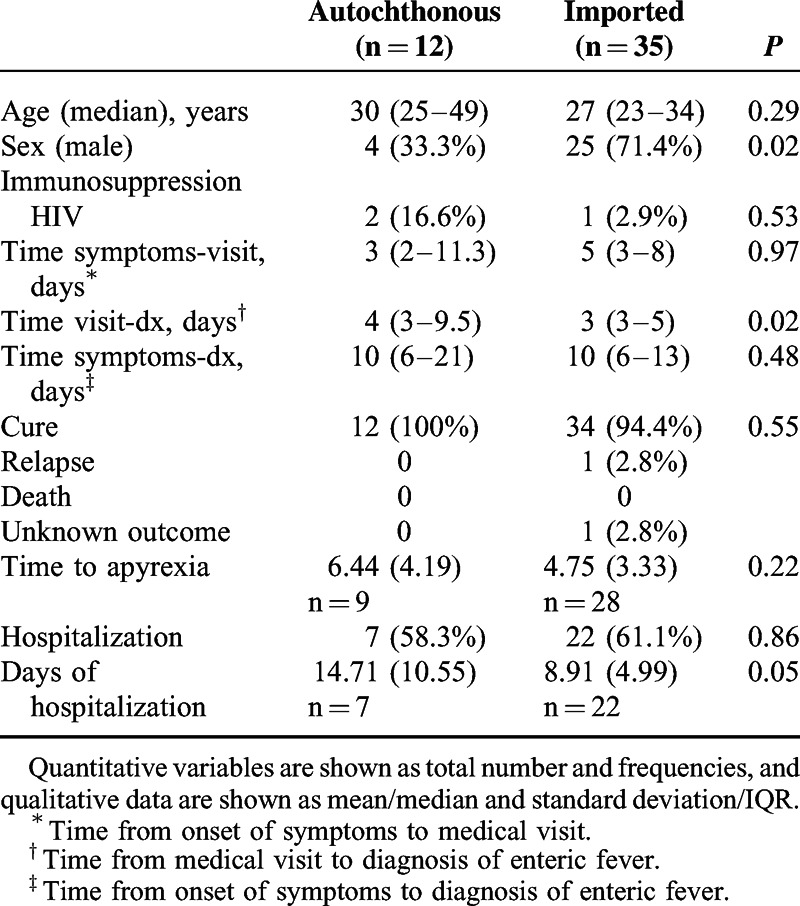
Patient Baseline Characteristics and Prognosis According to Travel History

The number of patients with imported enteric fever according to travel risk and geographic area is shown in Table 2. It is notable that 26 (70.3%) of the reported imported enteric fever patients came from Asia. Among travelers, the most frequently visited country was India (56.3%). Among VFRs, the most frequently visited country was Pakistan (50%). The vaccination rates among travelers and VFR were 60% and 33%, respectively.

**TABLE 2:**
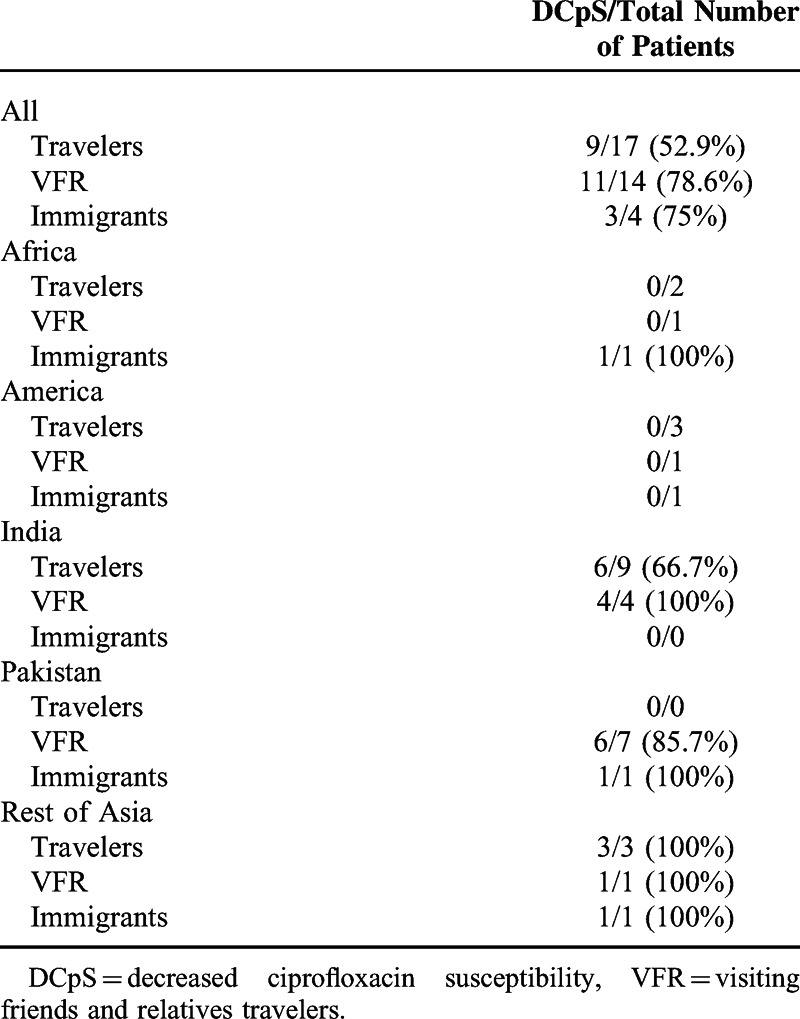
Number of Imported Enteric Fever According to Travel Risk, Geographic Area, and Quinolone Susceptibility

We identified 12 patients who acquired enteric fever in Spain. Four may have acquired the infection during an outbreak. Regarding their country of birth, 5 of the 12 (41.6%) patients were born abroad, but were living in Spain for at least 1 year before being infected. A brief summary of these patients is shown in Table 3 and the clinical and laboratory findings are shown in Table 4.

**TABLE 3:**
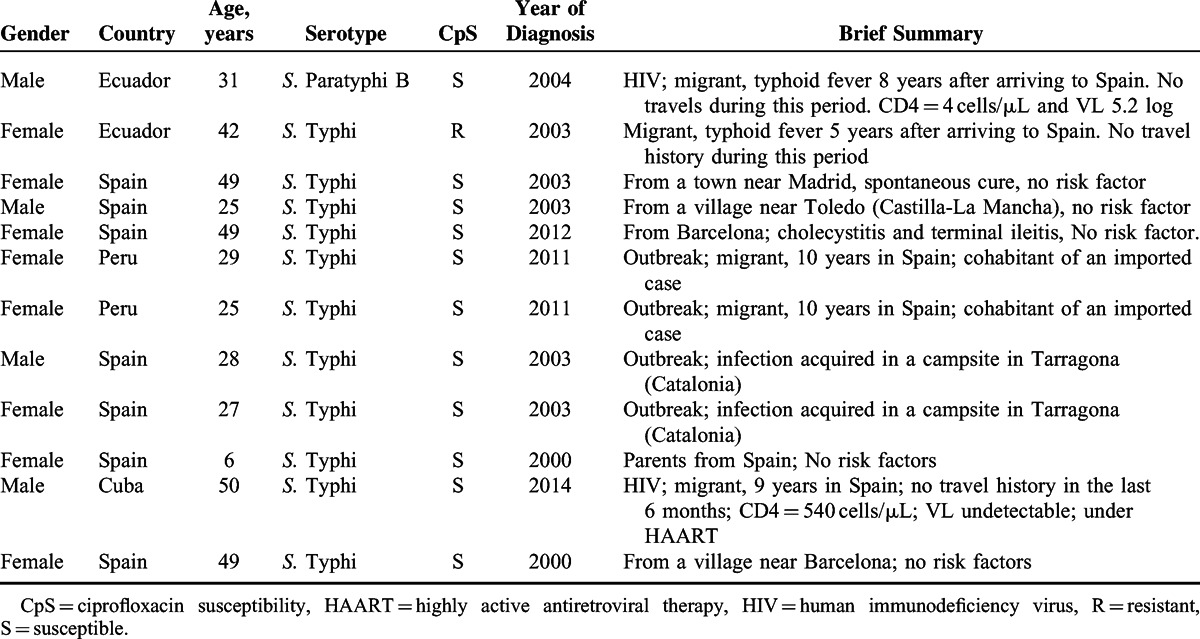
Brief Summary of Enteric Fever Acquired in Spain

**TABLE 4:**
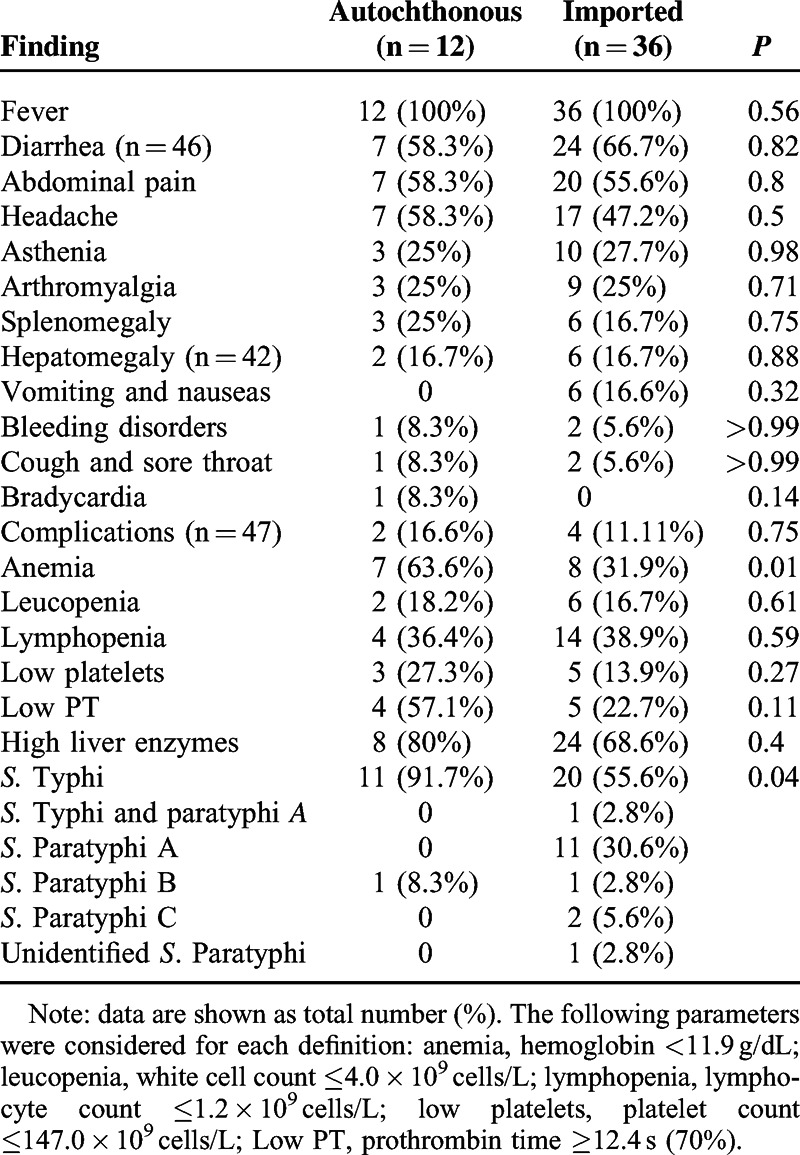
Episodes of Signs, Symptoms, Laboratory Findings, and Isolates According to Travel History

Most of the episodes of enteric fever were diagnosed by blood culture (44/48 patients) and by stool culture and systemic symptoms in the remaining 4 patients. Both blood and stool cultures were positive in 5 patients. *S. enterica* serotyping and drug-susceptibility test (DST) were available for all episodes and are shown in Tables 4 and 5, respectively.

**TABLE 5:**
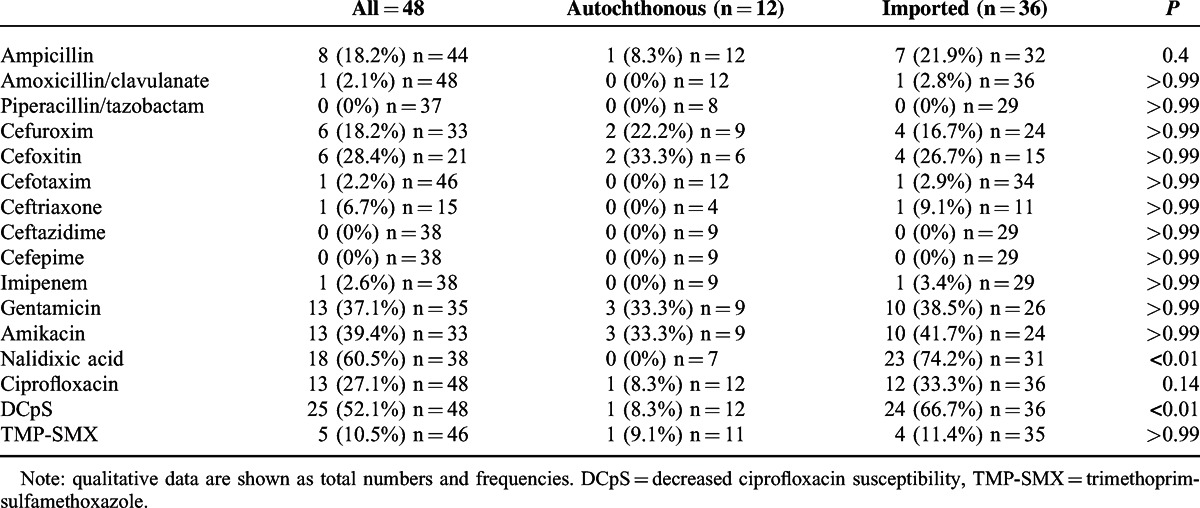
Episodes of *S. enterica* Antibiotic Resistance According to Travel History

Patients with travel history were significantly more likely to have decreased ciprofloxacin susceptibility (*P* = 0.001). All *S. enterica* isolated were susceptible to third-generation cephalosporins, except for 1 isolate that was identified as an extended-spectrum beta-lactamase (ESBL) producing *S.* Typhi strain.

Before a definitive diagnosis was made, 66.7% of patients in the autochthonous group and 60% of patients in the imported group were given empiric antibiotic treatment (*P* = 0.775). Amoxicillin was used in 35.5% of the patients, followed by ciprofloxacin (24.1%), third-generation cephalosporins (17.4%), azithromycin (10.3%), and piperacillin-tazobactam; penicillin and clarithromycin were used in only 1 patient each. Once the diagnosis was made and before DST was available, fluoroquinolones were chosen for treatment in 39.1% of the patients, third-generation cephalosporins in 39.1% and amoxicillin in 17.4%. Patients with an autochthonous infection received more third-generation cephalosporins as initial treatment, although this was not statistically significant (*P* = 0.12). The median treatment length was 12 days in both groups. The oral route was used as the initial treatment route in 30% of the autochthonous infection group and 52.8% of the imported infection group with no statistical significance (*P* = 0.202).

During the course of the treatment, 20 patients (55.6%) in the imported infection group had to change their initial treatment at least once. Twenty-eight treatment switches (some patients made >1 switch) were performed in the imported infection group. Their main reasons for switching were: sequential antibiotic therapy (SAT), which means converting patients from intravenous to oral medication, in 50% of patients; inefficacy (defined as persistence of fever or clinical signs consistent with persistent infection) in 32.1%; or based on the DST results in 14.3%. In the autochthonous infection group, 7 patients (58.3%) had to change their initial treatment at least once. Eight changes were made overall (1 patient changed twice). Their reasons for switching were: SAT in 50% of patients; DST results in 37.5%; and adverse effects in 12.5%. When SAT was evaluated, ciprofloxacin was the drug most used, followed by cefixime and amoxicillin.

Most patients in our study had a full recovery and were considered cured, except for 2 patients in the imported infection group: one who was lost during the follow-up and another who relapsed. When comparing hospitalization requirements, there was no significant difference seen between groups (autochthonous, 58.3% vs imported, 61.1%; *P* = 0.865); although the autochthonous infection group spent more days at hospital, no statistical difference was seen (autochthonous, 14.7 days vs imported, 8.91 days; *P* = 0.054). More information is shown in Table 1.

According to ciprofloxacin susceptibility, isolates from patients who had travelled to Asia were more likely to have decreased susceptibility to ciprofloxacin (odds ratio, 52.25; 95% confidence interval: 8.6–317.7). The 29.2% of patients with strains showing decreased susceptibility to ciprofloxacin received quinolones as initial treatment before DST was available. Table 6 shows more information regarding ciprofloxacin susceptibility.

**TABLE 6:**
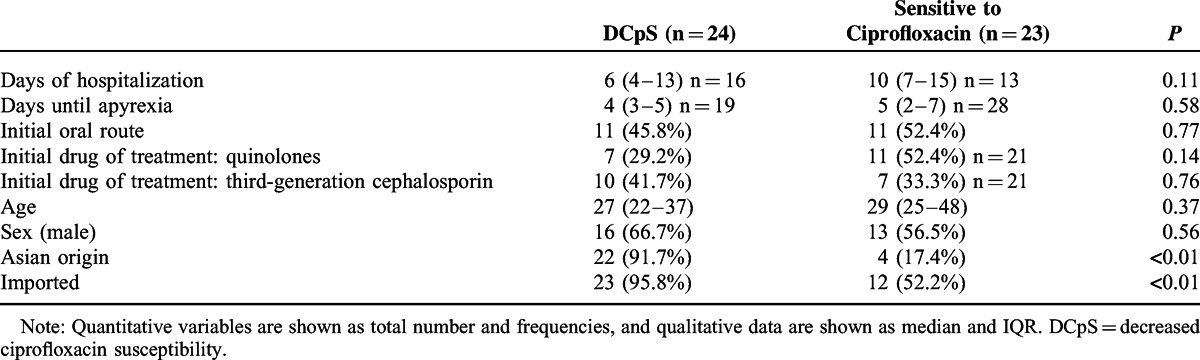
Patient Characteristics According to Ciprofloxacin Resistance

## DISCUSSION

Despite the decrease in enteric fever incidence in high-income countries in recent decades,^1,10^ international travel represents a risk factor for acquiring the disease. The disease is currently related to international trips, with the VFR subset at higher risk of acquiring the infection.^3^ Our study supports the hypothesis that enteric fever in Spain seems to be travel-related, and that patients with enteric fever mainly come from Asia. Thirty-five of 47 (74.5%) patients had enteric fever related to international travel and 26 of 35 (74.2%) patients with imported enteric fever infection had visited Asia. In concordance with other studies, our results showed a high proportion of travelers (48.6%), whereas VFR accounted for 40% of cases.^10,11^ Data from the United States and Europe revealed that 85% to 89% of typhoid fever cases were travel-related, with Asia being the main locus of infection.^1,11,12^ In a study of Israeli travelers, the attack rate for typhoid fever when travelling to the Indian subcontinent was 2.42 per 10,000 travellers-year.^10^ The countries with high incidence of typhoid fever in travelers show an even higher incidence in their local population, for example, typhoid incidence in 2 slums in Kolkata, India (all ages) and Karachi, Pakistan (2- to 15-year olds) were 214.2 and 451.7 per 100,000 person-year, respectively.^13^

Surprisingly, 12 patients (25.5%) did not have any travel history in recent years. Two of these patients were siblings and cohabitants of an imported infection case. One couple acquired the infection during their summer holidays in a camping area by a river delta. Considering this, these cases can be classified as outbreak cases. For the eight remaining cases, two were HIV patients and no risk factor or immunosuppressed condition could be demonstrated in six patients. Other studies also pointed out cases with unknown origin; however, we were surprised by the high proportion found in our study, mostly because Spain has a very low autochthonous enteric fever rate, which is similar to that of other Western European countries.^11,14,15^

Laboratory findings and clinical symptoms are in concordance with studies in other non-endemic countries.^14,16^ The results were unspecific and had low diagnostic utility. Classical signs such as rose spots were not described in any patient and relative bradycardia was described in only 1 patient. There were no complications resulting from perforation or neurological impairment, but 6 patients experienced complications, such as acalculous cholecystitis, ileitis, arthritis and demyelinating polyradiculoneuropathy, which have all been described previously.^17^

Concerning serovars of the enteric fever episodes, in a recent Israeli study, increasing incidence of *S*. Paratyphi was observed, whereas *S.* Typhi cases decreased. The reasons for this are unknown, although vaccination factors could be implicated, such as immunization coverage among travelers and the type of vaccine used.^10^ We found that 31 (66.7%) cases of enteric fever were because of *S.* Typhi, followed by 16 (33.3%) patients with *S*. Paratyphi serotypes. One patient had a combined infection with *S.* Typhi and *S*. Paratyphi. Since *S.* Typhi infection is clinically or analytically indistinguishable from *S*. Paratyphi infection,^18^ and vaccination practices against *S.* Typhi may increase *S*. Paratyphi infections, physicians attending returning travelers should be aware that vaccinated patients may present higher rates of *S*. Paratyphi infections.

The antimicrobial resistance of *S.* Typhi and *S*. Paratyphi is an important concern. The first-line treatment for enteric fever has changed over time because resistant strains have appeared.^3^ Nowadays, third-generation cephalosporins are the cornerstone of treatment. A report about typhoid fever in the United States during the period 1999 to 2006 found that 13% of *S.* Typhi strains were MDR. Nalidixic resistance was reported in 38% of isolates with 97% of those having decreased susceptibility to ciprofloxacin, and only 5 (0.2%) ciprofloxacin-resistant strains were identified.^1^ The data from our cohort showed only 6 patients with MDR, which may be explained by the decreased use of chloramphenicol, trimethoprim-sulphamethoxazole, and ampicillin as the first-line treatment options. Disturbingly, 60.5% of the isolates showed resistance to nalidixic acid. Furthermore, 27.1% of all isolates were ciprofloxacin-resistant or intermediate (9 of 13 were resistant and 4 were intermediate). In contrast to other reports, we did not find any ciprofloxacin-resistant or intermediate isolates that were susceptible to nalidixic acid.^12^

When analyzing imported enteric fever alone, nalidixic acid resistance increased until 74.2% and the ciprofloxacin resistance was 33.3%. *Salmonella* strains from patients returning from Asia have a high likelihood of having decreased susceptibility to ciprofloxacin. Classically, the Indian subcontinent has been considered as a region with high levels of quinolone resistance, while few cases have been reported from South Asia.^1,13,19^ We had 1 patient from Cambodia, 1 from Thailand, and 1 from the south of China and all of them were ciprofloxacin-resistant. Surveillance in this region should be encouraged and national programs empowered or created if absent to rapidly detect ciprofloxacin-resistant strains. One isolate from a traveler returning from the America was defined as an ESBL-producing *S.* Typhi strain; however, susceptibility to fluoroquinolones was preserved. A more detailed description can be found elsewhere.^20^

In our study, patients with imported enteric fever received mainly fluoroquinolones as the initial drug for treatment before DST was available. These treatments were ineffective in 29.2% of patients, in whom DST finally revealed a fluoroquinolone-resistant strain. It is likely that some physicians were aware of the increased fluoroquinolone resistance in Asia. Nevertheless, more training is needed among primary care physicians and emergency physicians to initiate third-generation cephalosporins when imported enteric fever is suspected, mainly if patients are returning from Asia. Although macrolides have been shown to be an effective treatment against typhoid fever, regardless of its fluoroquinolone resistance, they were not used as treatment in any of our patients.^21^

Outpatient management of typhoid fever is frequently performed in low-income countries when treating adult patients.^13^ European data suggest that the hospitalization rate is between 68.3% and 76.6%.^11,16^ Our study found that 56.25% of patients needed hospital admission, and the mean time of stay was 10.31 days, with a trend to longer stay in autochthonous patients. Hospitalization rates may reduce to similar rates as those of low-income countries if proper oral treatment was rapidly initiated and close follow-up by a specialist was offered. Reducing one hospitalization in Spain saves €2951 for the national health system.^15^

Our study has the limitations associated with a retrospective study. Data regarding symptoms and physical examination may show bias because some symptoms are not usually considered unless an enteric fever is suspected. Moreover, the incidence of enteric fever and the incidence of the decreased susceptibility of isolates cannot be calculated because no data from the total number of travelers or the total episodes of enteric fever are available. However, the findings concerning the reduced susceptibility to quinolone in patients coming from Asia highlight the need for further investigation and surveillance.

To summarize, our results highlight the emergence of quinolone-resistant *S.* Typhi and *S*. Paratyphi isolates in imported enteric fever, especially in travelers returning from Asia. This makes it mandatory to initiate treatment with an effective quinolone-alternative treatment (third-generation cephalosporins or azithromycin) in returning travelers and immigrants until DST is available. Prevention strategies such as pretravel counseling and immunization before travel may be beneficial, especially in countries with known quinolone-resistant strains.
